# St. Thomas's Hospital

**Published:** 1830-04

**Authors:** 


### ST. THOMAS'S HOSPITAL.

### *Retention of Urine from Stricture. Local application of Belladonna.*

John Schaaff, æt. sixty-one, a native of Saxony, residing in London, and where, for the last thirty years, he has been employed as a coppersmith and brazier, was admitted into Isaac's ward with retention of urine. His health has been generally pretty good; occasionally, however, he has been affected by colic. Has had stricture in the urethra for two years, and has been obliged at times to apply to a medical man, for the purpose of having his urine drawn off by a catheter.

The man came to the hospital about three o'clock on Sunday morning (February 24th), when the dresser found it impossible to introduce a catheter into the bladder, some blood following every attempt. After some time, however, he succeeded in passing a small-sized conical bougie, and some urine flowed, which greatly relieved the patient. The man refused to have any thing more done for him at that time, and he returned home to his bed.

At ten A.M. of the same day he returned, labouring under the like distress as at his first application. After some trouble a bougie was passed into the bladder, and a few ounces of urine followed on its being withdrawn. The stricture is situated about three inches from the orifice of the urethra. The man was now put into the warm bath, and a dose of castor oil given him. Mr. Tyrrell ordered him to take Tinct. Ferri Muriat. ♏xv.; Tinct. Opii ♏v. secundis horis. A bougie, rubbed over with Belladonna and oil, to be passed into the urethra. Soon after the man came from the bath, this was tried, and after two or three attempts the bougie (which was larger than those used before) readily passed the stricture, and the bladder was emptied of its contents. Poppy fomentation to be applied to the lower part of the abdomen and penis. About an hour after, another bougie was passed, and kept in the urethra two hours.

On the following day a bougie (conical pointed) was introduced two or three times, by which the stricture was sufficiently dilated to allow the urine to pass pretty freely.

The man was allowed to remain without any further regard to the stricture for a few days, for the purpose of allaying a considerable tumefaction of the penis; and on this subsiding, a bougie, simply oiled, could not be introduced beyond the stricture, and Belladonna was again resorted to, by which the irritability of the stricture seemed to be allayed, and the bougie passed into the bladder.

The patient now passes his urine whenever he feels a desire, and the present treatment adopted is merely attending to the secretions of the alimentary canal, and the introduction of a bougie daily.*

* Ibid.

